# A Case Report of Generalization of Localized Actinic Granuloma, an Unusual Diffuse Presentation Not Responding to Tofacitinib

**DOI:** 10.1002/ccr3.70618

**Published:** 2025-07-07

**Authors:** Saman Al‐Zahawi, Alireza Ghanadan, Fatemeh Saberi

**Affiliations:** ^1^ Department of Dermatology, Razi Hospital Tehran University of Medical Sciences (TUMS) Tehran Iran; ^2^ Department of Dermatopathology, Razi Hospital Tehran University of Medical Sciences (TUMS) Tehran Iran

**Keywords:** actinic granuloma (AG), annular elastolytic giant cell granuloma (AEGCG), cyclosporine, granuloma annulare (GA), tofacitinib

## Abstract

Actinic granuloma (AG), a rare and idiopathic skin condition primarily affecting middle‐aged and elderly individuals, is known for its persistent, asymptomatic annular plaques appearing on sun‐exposed areas. This report details the case of a 48‐year‐old diabetic male farmer with a rare presentation of generalized AG. The patient's lesions were unusually widespread, affecting sun‐exposed sites, and failed to respond adequately to tofacitinib therapy.

**Summary**

- Actinic granuloma is notoriously difficult to treat, often resisting all therapies, including newer medications like JAK inhibitors.

## 1 Introduction

Actinic granuloma (AG) is a rare idiopathic dermatosis of middle‐aged and elderly patients, characterized by asymptomatic annular plaques on chronically sun‐exposed sites of the body with a tendency to persist despite therapy [1]. A precise biopsy of the raised border will show engulfed elastic fibers in multinucleated giant cells and an absence of elastic fibers in the center of the annular lesions [2]. Diabetes mellitus, sarcoidosis, lymphoma, and leukemia are rare diseases that have been reported in association with AG [3]. Due to its rarity and the lack of a clear consensus on effective treatment, AG presents a therapeutic challenge. While a photoinduced condition, it has paradoxically been reported to respond to Psoralen + Ultaviolet A (PUVA). Other treatment options include topical or intralesional corticosteroids, dapsone, retinoids, and cyclosporine [1].

## 2 Case Presentation/Examination

A 48‐year‐old diabetic male farmer presented with diffuse skin lesions initially confined to sun‐exposed areas. Two years prior, he developed a few small (< 2 × 2 cm) lesions on his face, which were partially treated with short courses of hydroxychloroquine and intralesional corticosteroids. Recently, he experienced a sudden eruption of numerous larger, annular lesions on his face, neck, upper chest, and distal extremities.

**FIGURE 1:**
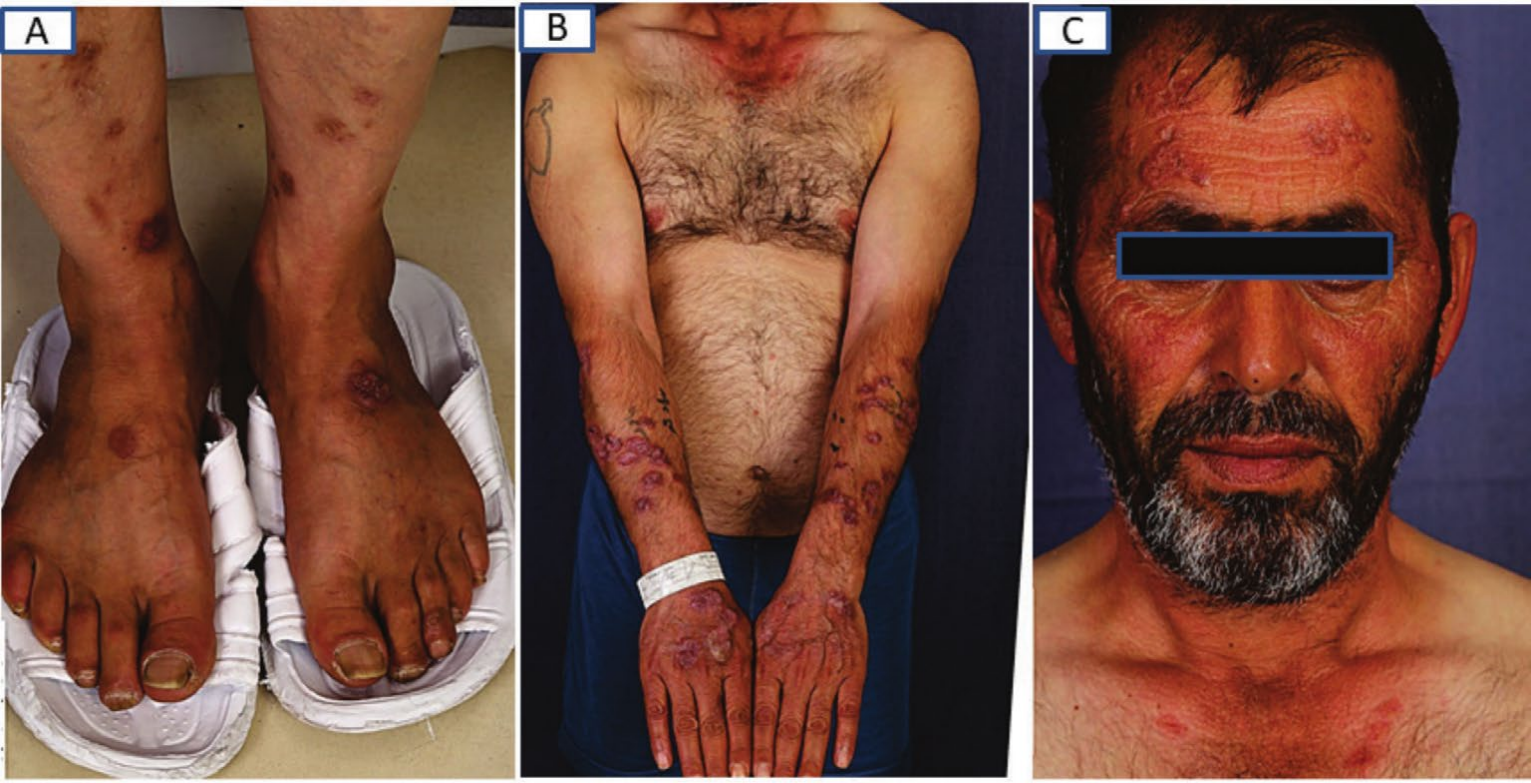
Mutiple erythematous‐brown colored annular plaques with raised border and central clearing in lower extremity (A), upper extremity and V shaped region of the trunk (B), and face (C).

**FIGURE 2:**
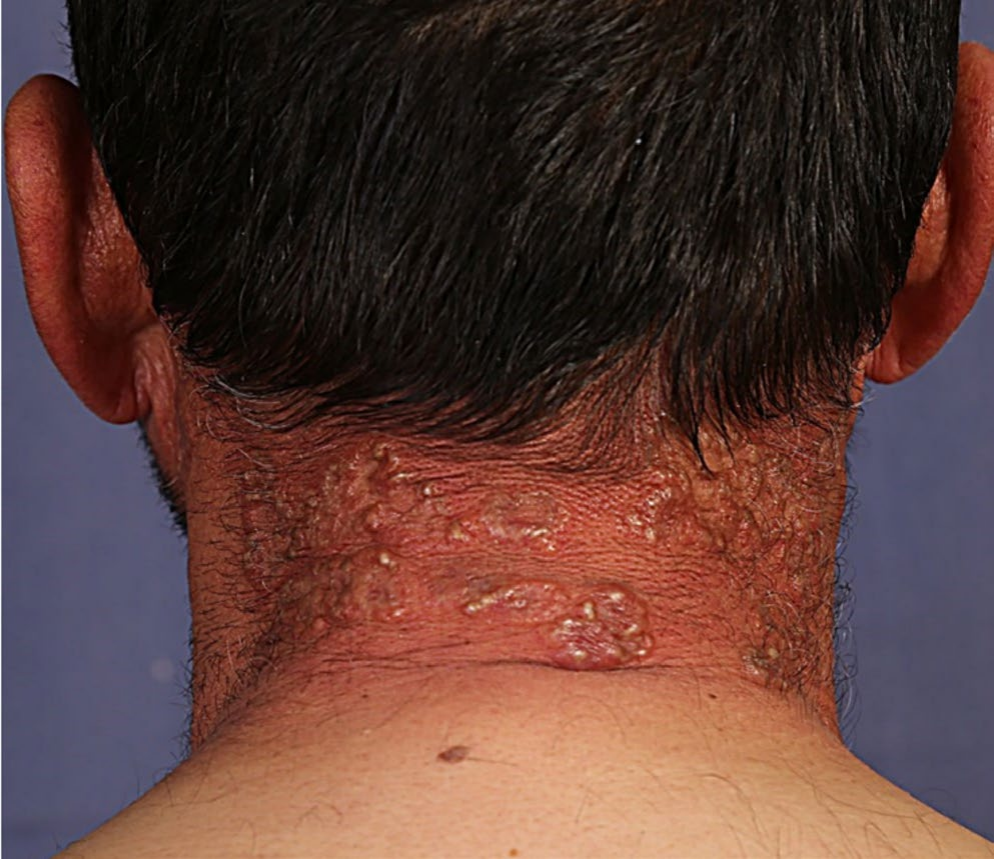
Coalescing of multiple lesions to form a serpiginous plaque of more than 10 cm.

Physical examination revealed approximately 50 annular lesions with raised borders, ranging from 1 to 5 cm in diameter, distributed across sun‐exposed areas of the head, upper and lower extremities (Figure 1A–C). On the neck and dorsal hands, some lesions had coalesced into larger, serpiginous plaques exceeding 5 cm (Figure 2).

### 2.1 Differential Diagnosis, Investigations, and Treatment

**FIGURE 3:**
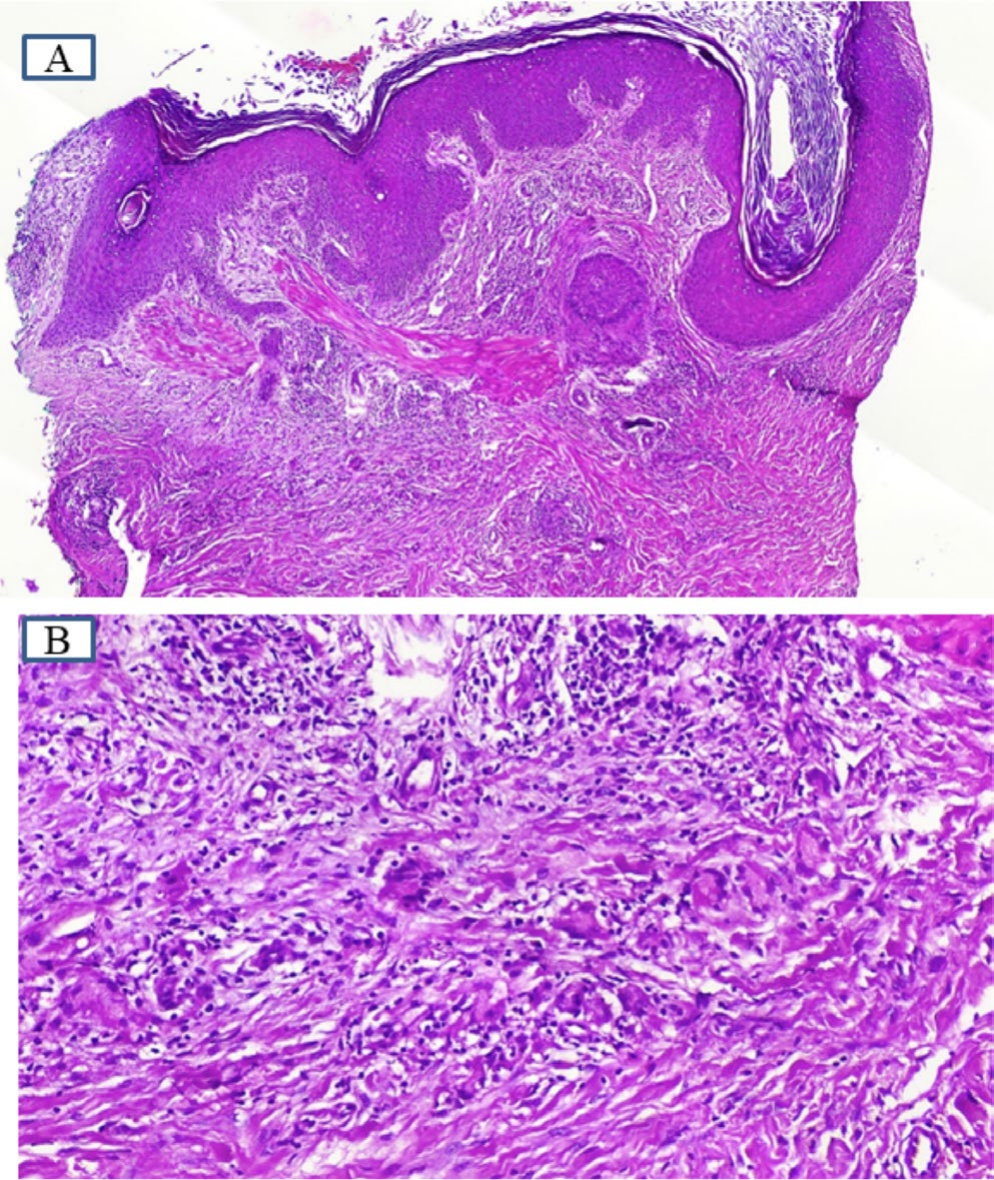
Skin punch biopsy from the border of right forearm lesion showing mild hyperkeratosis, irregular acanthosis, and follicular hyperkeratosis associated with hyperplastic follicular infundibulum and elongated rete ridges (A). There are perifollicular infiltrates of lymphohistiocytes and some giant cells, admixed with some plasma cells and fragments of elastin in giant cells (B).

Initial pathology results from 2 years ago indicated granuloma annulare. However, the lesions proved resistant to treatment with intralesional corticosteroids, hydroxychloroquine, retinoids, and PUVA and remained localized to sun‐exposed areas. The sudden appearance of numerous new lesions further complicated the diagnosis, necessitating a second biopsy. This subsequent biopsy, performed on the right forearm, revealed AG, revising the initial granuloma annulare diagnosis (Figure 3A,B). Only HbA1c and fasting blood sugar were elevated in the biochemical analysis.

The patient was initially treated with oral tofacitinib for 4 months due to a nationwide shortage of cyclosporine. This treatment proved ineffective in halting the disease's progression. Following the availability of cyclosporine, a regimen of 200 mg daily was initiated, along with sun avoidance and regular follow‐up appointments.

### 2.2 Outcome and Follow‐Up

We present an unusual case of generalized AG in a 48‐year‐old diabetic male farmer. The patient's lesions, which spread to sun‐exposed sites, showed a poor response to tofacitinib. Unfortunately, the patient was lost to follow‐up, preventing evaluation of the subsequent cyclosporine treatment.

## 3 Discussion

Actinic elastolytic giant cell granuloma and AG are synonymous terms for annular plaques appearing on sun‐exposed skin, characterized pathologically by granulomatous inflammation with multinucleated giant cells and elastophagocytosis. It has been hypothesized that these lesions result from an immune response against damaged elastic fibers in areas with prolonged sun exposure. Although AG tends to have a persistent course, spontaneous resolution may occur without scarring, but with possible dyspigmentation [4]. Despite the usual role of not leaving a scar behind, Gavioli et al. reported scarring and open comedones at the site of resolved lesions [5].

Unlike the typical annular form, the papular variant of AG can occur on non‐sun‐exposed skin, has a shorter duration, an earlier onset, and a stronger association with systemic diseases [6, 7]. While the mean age of classic AG is 60 years with a female predilection, patients as young as a 21‐year‐old with conjunctival AG have been reported [8, 9]. Conjunctival AG appears as red eye masses and may be confused with pterygium. In addition to the conjunctiva, the palm is another unusual site of involvement [10]. Our patient had a striking photo‐distributed pattern of lesions, highlighting the role of ultraviolet light in the pathogenesis of AG. AG has also been reported in non‐photo‐related conditions such as after cardiac pacemaker implantation [11].

While AG has been linked to various conditions, including sarcoidosis, diabetes, leukemia, and lymphoma, diabetes mellitus is the most commonly reported association, as seen in our case [12, 13]. Sonal et al. emphasized the association of AG with temporal arteritis and close evaluation of patients with AG to exclude this devastating condition [14].

Clinically, AG can resemble granuloma annulare. However, the absence of mucin deposition (a hallmark of granuloma annulare), the lack of collagen changes, and the loss of elastic fibers (demonstrated by Verhoeff‐Van Gieson staining) distinguish AG pathologically from granuloma annulare. The lack of lipid deposition aids in differentiating the granulomatous changes of AG from necrobiosis lipoidica, but very rarely, the pathological diagnosis of AG may be confounded when there are overlapping findings with granuloma annulare and necrobiosis lipoidica. Although AG may resolve spontaneously, treatment is usually needed as it tends to persist and very rarely progress to a more generalized pattern.

For localized AG, treatment typically begins with topical or intralesional corticosteroids. For more generalized lesions, both successful treatment and induction of AG have been reported with pulsed dye laser [15, 16]. Also, resolution of AG lesions has been reported with acitretin and topical tretinoin [3, 17]. Recently, the successful treatment of AG has been reported with 6 months of treatment with doxycycline [18]. Lastly, successful treatment of AG with PUVA, dapsone, cyclosporine, and chloroquine has been reported [13].

When the patient presented to our dermatological center, cyclosporine was unaffordable, and tofacitinib in a dose of 10 mg/day was given to the patient. JAK inhibitor was chosen based on the new studies that show the implication of Janus kinase–signal transducer and activator of transcription (JAK–STAT) pathway in the pathogenesis of granuloma formation and its possible effect in patients with AG [19], in addition to recent reports of treating patients with AG with baricitinib [20]. Unlike the evidence previously discussed, a 4‐month course of tofacitinib was ineffective in halting the disease progression.

## 4 Conclusion

Actinic granuloma tends to persist and poorly respond to the available modalities of treatment. Very rarely, the clinical picture of the plaque type AG may change from localized lesions in one anatomical site to multiple lesions in different anatomical locations confined to the sun‐exposed sites. To our knowledge, tofacitinib is not effective in halting the progression of generalized AG.

## Author Contributions

**Saman Al‐Zahawi:** writing – original draft, writing – review and editing. **Alireza Ghanadan:** visualization. **Fatemeh Saberi:** writing – original draft, writing – review and editing.

## Consent

Written informed consent was obtained from the patient to publish this report in accordance with the journal's patient consent policy.

## Conflicts of Interest

The authors declare no conflicts of interest.

## Data Availability

The authors elect to share data per request.
